# Power-law scaling in intratumoral microbiota of colorectal cancer

**DOI:** 10.1186/s13099-024-00631-x

**Published:** 2024-07-07

**Authors:** Nikolas Dovrolis, Maria Gazouli, François Rigal, Robert J. Whittaker, Thomas J. Matthews, Konstantinos Georgiou, George Theodoropoulos, Kostas A. Triantis

**Affiliations:** 1 Department of Basic Medical Sciences, Laboratory of Biology, Medical School, National and Kapodistrian University of Athens, Athens, 11527 Greece; 2 Institut Des Sciences Analytiques et de Physico Chimie pour L’environnement et les Materiaux, CNRS - Université de Pau et des Pays de l’Adour - E2S UPPA, UMR5254, Pau, 64000 France; 3 CE3C – Centre for Ecology, Evolution and Environmental Changes/Azorean Biodiversity Group, Faculty of Agricultural Sciences and Environment, CHANGE – Global Change and Sustainability Institute and Universidade dos Açores, Angra do Heroísmo, Açores, PT-9700-042 Portugal; 4 School of Geography and the Environment, University of Oxford, Oxford, UK; 5 Center for Macroecology, Evolution and Climate, GLOBE Institute, University of Copenhagen, Copenhagen, Denmark; 6 GEES (School of Geography, Earth and Environmental Sciences, Birmingham Institute of Forest Research, University of Birmingham, Birmingham, UK; 7 1st Department of Propaedeutic Surgery, Medical School, National Kapodistrian University of Athens, Hippocratio Hospital, Athens, 11527 Greece; 8 Department of Ecology and Taxonomy, National & Kapodistrian University of Athens, Athens, 11527 Greece

**Keywords:** Intratumoral microbiome, Cancer, Island species–area relationship

## Abstract

It has recently been proposed that the study of microbial dynamics in humans may gain insights from island biogeographical theory. Here, we test whether the diversity of the intratumoral microbiota of colorectal cancer tumors (CRC) follows a power law with tumor size akin to the island species-area relationship. We confirm a direct correlation between the quantity of Amplicon Sequence Variants (ASVs) within CRC tumors and tumor sizes, following a (log)power model, explaining 47% of the variation. Understanding the processes involved, potentially through the analogy of tumors and islands, may ultimately contribute to future clinical and therapeutic strategies.

**Supplementary Information:**

The online version contains supplementary material available at 10.1186/s13099-024-00631-x.

Power laws are found in numerous natural, social, and artificial systems, phenomena [1–4] and are particularly prevalent in biology, ecology, and biogeography, demonstrating that evolutionary, ecological, and physiological constraints apply consistently to genomes, cells, organisms, communities, and ecosystems across vast orders of scale [1, 5–8]. One of the best-established ecological laws is the island species–area relationship (ISAR), wherein the number of species (‘species richness’) occurring per island increases per island area. This pattern has been substantiated by studies spanning diverse spatiotemporal scales and taxa, including the microbiome [9, 10], and has been found to be best expressed as a power function which, when linearized via logarithmic transformation, is given by the equation logS = logc + zlogA, where S denotes species richness, A represents area, and c and z are fitted parameters. The slope of the ISAR, i.e. the z parameter, is often considered informative of the dominant processes of species addition [11, 12].

The seminal paper describing the Human Microbiome Project [13] references island biogeography as a driving force behind our ability to study and understand spatiotemporal microbial dynamics, while a recent study proposes a positive scaling relationship between human height and gut microbiome alpha-diversity across two large, independent cohorts, controlling for a wide range of relevant covariates [14]. Notwithstanding that cancer tumor dynamics may depend on a variety of variables spanning cell proliferation inclination and genomics [15] to microbial interactions and metagenomics [16], recent research has proposed that tumors can usefully be considered as island-like systems [17, 18]. Here, we explore this analogy by testing for an ISAR-like relationship between the microbiota, residing inside the tumor as a first step in applying island biogeography principles and theories to understanding and predicting tumor dynamics. These intratumoral microbiota may contribute to shaping the tumor microenvironment by influencing the immune environment, inflammation, and metabolic patterns of the tumor [19], hence they may provide novel ways to study cancer prognosis, development, and therapy [20].

Using a novel cohort of tumors from colorectal cancer (CRC) patients (*n* = 27), a cancer type highly connected to microbial dysbiosis [21], we test for a potential relationship between the size of a tumor (measured as its diameter) and the composition of bacteria residing within the tumor. These bacteria are identified by analyzing their unique DNA sequences, called Amplicon Sequence Variants (ASVs), which act as the “species units” for this study. We hypothesize that the intratumor microbiota populations across patients will mirror the ISAR, by increasing in diversity as the tumor’s size increases, following a power law model. The power model is the most frequently preferred, both in describing ISARS and as a basis for the development of species diversity theories [11, 12].

Using a linearized power model with a logarithmic transformation of the variables (the log-log model), we identified a significant positive species–area relationship (Fig. 1A, Table S2): tumor size explaining 47% of the number of ASVs identified within CRC tumors. Rarefaction curves indicated that all tumors were sufficiently sequenced to capture total ASV within-sample richness (reached an asymptote, Figure S1). We are therefore confident that the aforementioned results are robust. However, as a sensitivity test, the analysis was rerun using rarefied species richness and the ISAR remained significant (Fig. 1B, Table S2). For all regression models, no specific deviations from residual normality and homoscedasticity and no outliers were detected (Table S2). Further sensitivity tests were run to evaluate the model’s predictive power using a *k*-fold cross-validation procedure. This analysis revealed a correlation between the observed and the predicted log_10_ ASVs (Pearson’s Correlation Coefficient = 0.65) with a 95% confidence interval of [0.51; 0.79] calculated across 1000 simulations (see Fig. 1C). Using rarefied richness, a mean Pearson’s Correlation Coefficient of 0.55 was obtained with a 95% confidence interval of [0.36; 0.70]. These analyses support the robustness of our findings and indicate that the relationships described here have predictive power, i.e. they may extend to other tumors.

While we caution that (i) using diameter alone to measure tumor size is a potential limitation, and (ii) varied individual ages and cancer stages are potential confounding factors that we did not analytically account for, the strength of the observed pattern across diverse tumors suggests further exploration of the analogy with island dynamics to hold potential.

The similarity in the value of the slope of the diversity–tumor size relationship with patterns found in oceanic islands/archipelagos [11, 12] is intriguing. While we cannot assert that tumor ontogeny resembles that of oceanic islands, our findings serve as a starting point for further investigations into the commonalities between tumor growth and the colonization and diversification dynamics observed in island ecosystems. Studies have shown that the origins of intratumoral microbiota may stem from various pathways like proximity to the mucosal barrier, colonization from neighboring normal tissues enabled by the immunosuppressive and hypoxic conditions, or hematogenous dissemination where microorganisms from other sites invade the tumor via compromised blood vessels [20, 22]. Parallels can also be drawn to the island theory in terms of the effects environmental heterogeneity has on species richness, with the more diverse islands, usually the larger ones, hosting higher richness [11, 12]. Similarly, recent studies have found that intratumoral microbiota are not randomly distributed but rather highly organized into micro-niches, with functions related to immune and epithelial cells, thereby contributing to the progression of cancer [22], since larger tumors, often characterized by irregular morphology, potentially harbor more of these niches [23]. Thus, tumors’ morphology can be a potential explanation for the pattern reported herein.

In conclusion, our findings not only contribute to our understanding of tumor biology but also concur with the plea for interdisciplinary exploration by drawing parallels between tumor growth patterns and well-established ecological principles [24]. The study of the intratumoral microbiota and their dynamics holds potential to significantly impact future clinical and therapeutic endeavors [25]. Further research is warranted to unravel the mechanistic underpinnings of the observed similarities and explore the potential implications for both oncology and ecology.

**Fig. 1:**
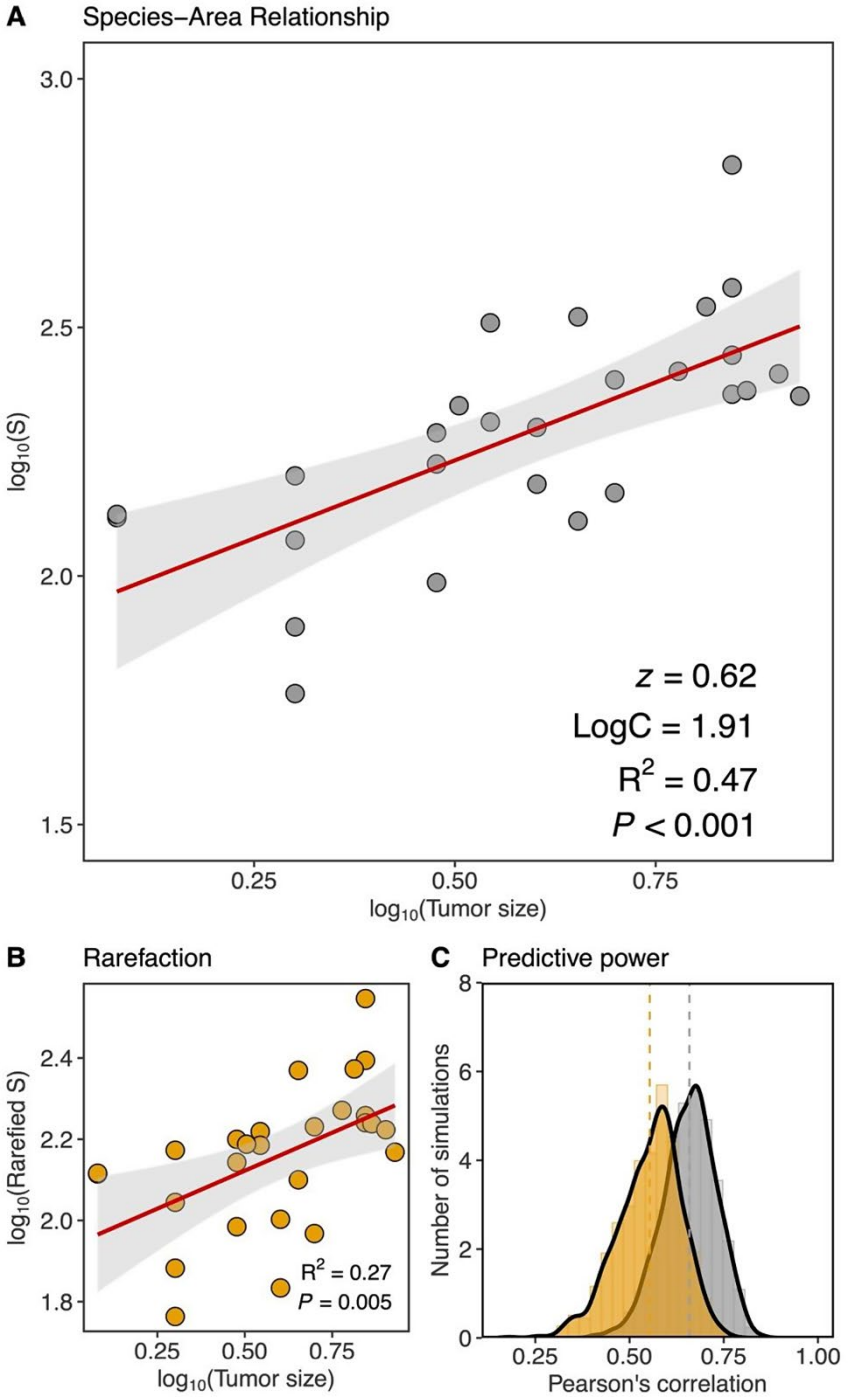
(**A**) Relationship between the log_10_-species richness (Number of Amplicon Sequence Variants, ASVs) and log_10_-tumor size, determined using Ordinary Least Square regression; the parameter *z* and LogC of the model as well as the associated R^2^ and the P-values of the F-statistic are given. More details on the OLS are given in Table S2. (**B**) Relationship between the log_10_ of rarefied species richness (Number of Amplicon Sequence Variants, ASVs) and the log_10_-tumor size (R^2^ and P-values of the F-statistic are given). Rarefaction was implemented with 1000 iterations. See Table S2 for details of the OLS. (**C**) The predictive power of the relationship in (**A**) and (**B**) evaluated using repeated *k*-fold cross-validation approach with 1000 simulations. The distribution of Pearson’s correlation between observed and predicted species richness values obtained for the 1000 simulations is given for both rarefied (yellow) and non-rarefied species richness (grey). Dashed lines indicate the mean correlation

## Methods

### Patient samples & DNA extraction

In this study, resected tumor samples from 27 CRC patients treated in the Colorectal Unit of the First Propaedeutic Department of Surgery, Hippocration General Hospital, Athens, Greece were analyzed. Samples were subsequently frozen in liquid nitrogen for one hour and kept at -80℃ before processing. All tumors selected ranged from 1.2 cm to 8.5 cm in diameter and were histologically inspected to validate the diagnosis. The longest length of the tumor in the tissue removed during surgery is reported as the tumor size. DNA extraction was performed using Macherey Nucleospin Tissue (MNT, MACHEREY-NAGEL) according to the manufacturer’s instructions. Sample metadata includes tumor size, patient age and sex as well as histological tumor grades, and can be found in Supplementary file S3.

### Sequencing and read processing

Sequencing was carried out by Eurofins Genomics Europe Sequencing GmbH (Jakob-Stadler-Platz 7, 78,467, Constance, GERMANY) on an Illumina MiSeq platform producing paired-read samples of 300 bp read length based on the V3-V4 amplicons. Raw sequences were quality controlled using CUTADAPT v2.7 [26] and used as input to QIIME2 v.2023.5 [27] on which they were denoised and clustered into ASVs (Amplicon Sequence Variants) using DADA2 [28]. ASV counts, sample metadata and taxonomy assignment tables are provided in Supplementary file S3.

### Statistical analyses

All of the analyses were undertaken in R [29, 30]. Several different mathematical models with different forms for describing ISARs have been proposed but recent papers have underlined that the power model provides the best fit to the ISAR in most cases [11, 12]. Traditionally, the power model is linearized by logarithmic transformations of species richness S and area A using the equation logS = logC + *z*logA, with *z* the slope of the resulting log–log relationship, and logC the intercept [11, 12]. Here, we employed the log–log linear modeling approach (using log10) to investigate the relationship between the number of Amplicon Sequence Variants (ASVs) detected per sample and tumor size. Prior to analysis, the completeness of each of the 27 samples was assessed using a rarefaction curve. As all rarefaction curves reach an asymptote, completeness was considered maximum for all samples and, consequently, with the number of ASVs directly comparable between tumors. However, as a sensitivity test, we refitted the ISAR log-log model using rarefied ASV richness using as sample size, the smallest library size encountered in our data i.e. 4231 sequences. Rarefaction was implemented using the R package *metagMisc* [31] with the function *phyloseq_mult_raref_div* and the number of iterations set to 1000. For each model, three regression diagnostics were implemented, namely the Shapiro-Wilk test for the normality of the residuals, the Pearson’s correlation between the fitted values and squared residuals for the homogeneity of the residuals, and the outlier t-test based on the Studentized residuals implemented with the function *outlierTest* in the *car* [32] R package. To assess the generality of our results, we adopted a repeated *k*-fold cross-validation approach whereby we randomly partitioned the datasets into three equal components (*k* = 3). For each partitioning, we put aside one component as the test data (9 samples) and fitted the log–log model to the remaining two components (the training data, 18 samples), and used the resultant model to predict the values of log_10_–species richness in the test data. The process was repeated for the three distinct combinations of training and test data. The predictive power of the log–log model was then assessed based on the Pearson’s correlation calculated between the predicted and observed values of the test data and subsequently averaged across the three combinations. This 3-fold cross-validation process was then repeated 1000 times. This procedure was also implemented for the model fitted with rarefied species richness.

### Electronic supplementary material

Below is the link to the electronic supplementary material.

Supplementary Material 1

Supplementary Material 2

Supplementary Material 3

## Data Availability

No datasets were generated or analysed during the current study.
