# Supplementary material for: Power-law scaling in intratumoral microbiota of colorectal cancer
Source: Gut Pathog. 2024 Jul 7;16:34. doi: 10.1186/s13099-024-00631-x (PMC11229225; doi:10.1186/s13099-024-00631-x)
Supplement: Supplementary file 1 — Supplementary Material 1 [file 13099_2024_631_MOESM1_ESM.docx]

**Table S2**. Results of the Ordinary Least Square regressions (OLS) between log_10_-species richness and log_10_ of tumor size tested before and after rarefaction. The slope *z* and the intercept LogC of the model are given as well as the R^2^, the *F*-statistic and its associated P-values (*P*). For both models, three regressions diagnostics were implemented: the Shapiro-Wilk test for the normality of the residuals, the Pearson’s correlation between the fitted values and squared residuals for the homogeneity of the residuals, and the outlier t-test based on the Studentized residuals. The P-values (P) of the test are shown for the normality and homogeneity tests, while for the outlier t-test, the lowest Bonferroni-corrected P-value obtained on all data points is shown. If this P-value is greater than 0.05, this means that no data points were identified as outliers.

|  | Model | |
| --- | --- | --- |
| Ordinary Least Square Regression | S | Rarefied S |
| *Z* | 0.627 | 0.374 |
| LogC | 1.919 | 1.935 |
| R^2^ | 0.469 | 0.276 |
| *F*-statistic | 22.060 | 9.525 |
| *P* | <0.001 | 0.005 |
| Regression diagnostics |  |  |
| *P* for Shapiro-Wilk test Normality of the residuals | 0.996 | 0.696 |
| *P* for Pearson’s Correlation for the homogeneity of the residuals | 0.672 | 0.664 |
| *P* for Outlier t-test | 0.417 | 0.682 |
