# Supplementary material for: Power-law scaling in intratumoral microbiota of colorectal cancer
Source: Gut Pathog. 2024 Jul 7;16:34. doi: 10.1186/s13099-024-00631-x (PMC11229225; doi:10.1186/s13099-024-00631-x)
Supplement: Supplementary file 2 — Supplementary Material 2 [file 13099_2024_631_MOESM2_ESM.docx]

**
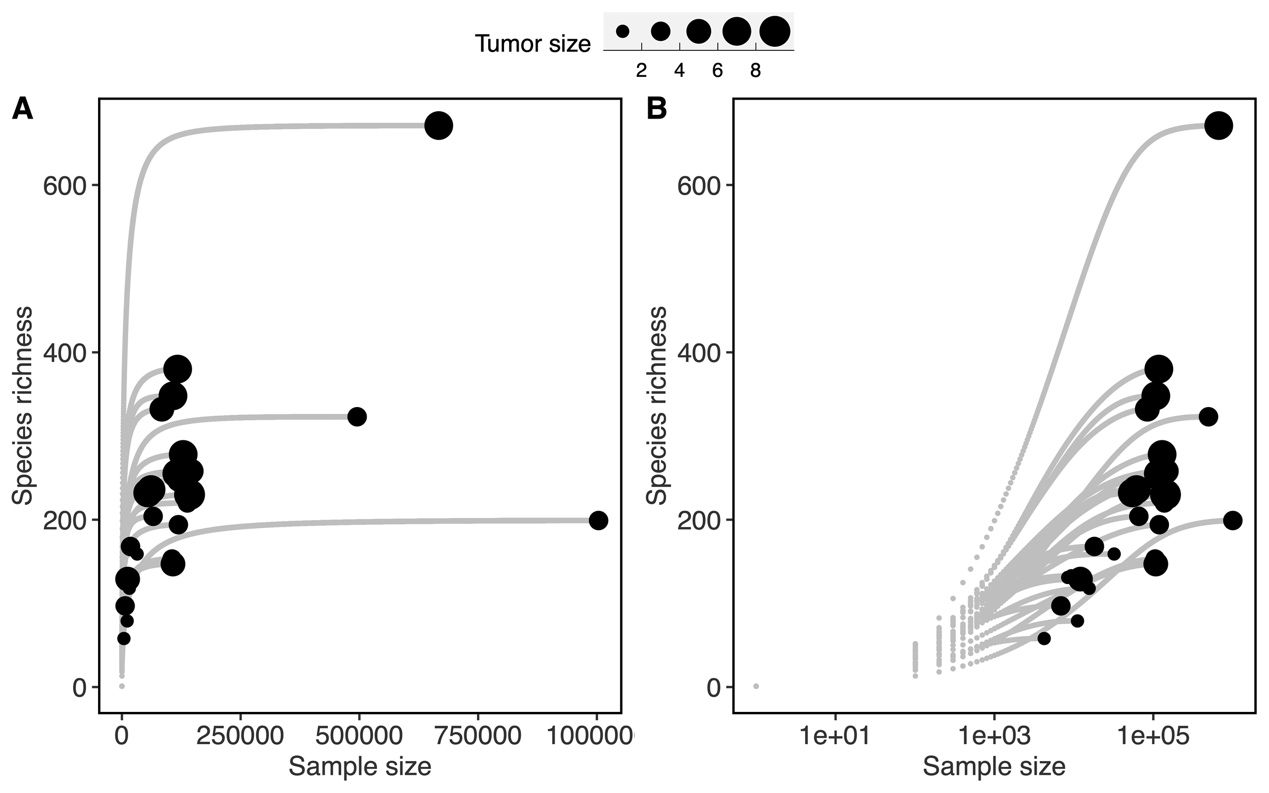
**

**Figure S1.** Rarefaction curves for the 27 samples. The curves are given in their standard version (A) and with sample size in a logarithmic scale to facilitate the visualization of the results for the samples with the smaller sizes (B). For each curve and in both panels, a point is added at the end of the curve with a size proportional to the tumor size. The point sizes used for these proportions only serve visualization purposes and encompass all actual tumor size intervals.
